# Novel Endophytic *Pseudescherichia* sp. GSE25 Strain Significantly Controls *Fusarium graminearum* and Reduces Deoxynivalenol in Wheat

**DOI:** 10.3390/toxins15120702

**Published:** 2023-12-15

**Authors:** Meiling Gao, Mohamed F. Abdallah, Minggui Song, Yiqian Xu, Daiyuan Sun, Ping Lu, Jianhua Wang

**Affiliations:** 1 National Engineering Research Center of Seafood, School of Food Science and Technology, Dalian Polytechnic University, Dalian 116034, China; gaoml@dlpu.edu.cn; 2 State Key Laboratory of Crop Stress Resistance and High-Efficiency Production, College of Plant Protection, Northwest A&F University, Xianyang 712100, China; smg@nwafu.edu.cn (M.S.);; 3 Department of Food Technology, Safety and Health, Faculty of Bioscience Engineering, Ghent University, Coupure Links 653, 9000 Gent, Belgium; mfathiabdallah@gmail.com; 4 Department of Forensic Medicine and Toxicology, Faculty of Veterinary Medicine, Assiut University, Assiut 71515, Egypt; 5 The Key Laboratory for Quality Improvement of Agricultural Products of Zhejiang Province, College of Advanced Agricultural Sciences, Zhejiang A&F University, Hangzhou 311300, China; 6 Institute for Agri-Food Standards and Testing Technology, Shanghai Academy of Agricultural Sciences, Shanghai 201403, China

**Keywords:** wheat, *Pseudescherichia* sp., fusarium heading blight, deoxynivalenol, *Fusarium graminearum*, biocontrol

## Abstract

Fusarium heading blight (FHB) is a devastating disease in wheat, primarily caused by field invasion of *Fusarium graminearum*. Due to the scarcity of resistant wheat varieties, the agricultural sector resorts to chemical fungicides to control FHB incidence. On the other hand, biocontrol represents a promising, eco-friendly approach aligned with sustainable and green agriculture concepts. In the present study, a bacterial endophyte, *Pseudescherichia* sp. (GSE25), was isolated from wheat seeds and identified through complete genome sequencing and phylogenetic analysis. In vitro testing of this endophytic strain demonstrated strong antifungal activity against *F. graminearum* PH-1 by inhibiting spore germination, suppressing germ tube growth, and causing cell membrane damage. Under field conditions, the strain GSE25 significantly reduced the FHB incidence and the associated deoxynivalenol mycotoxin accumulation by over 60% and 80%, respectively. These findings highlight the potential of the isolated bacterial endophyte *Pseudescherichia* sp. GSE25 strain as a biocontrol agent in protecting wheat from FHB-caused *F. graminearum*. This is the first report showing a biocontrol effect of *Pseudescherichia* sp. a strain against phytopathogens.

## 1. Introduction

Fusarium head blight (FHB) is a common devastating fungal disease in wheat, primarily caused by *Fusarium graminearum* [1]. This disease is not only a yield-limiting factor in wheat but also attacks wheat heads, accumulating several mycotoxins. Among these mycotoxins, deoxynivalenol (DON) poses a significant risk to both humans and animals upon exposure [2]. While returning straw to the farmland ecosystem is a common practice to boost soil organic matter content, excessive straw can slow its decomposition rate. This situation may elevate the incidence of soil-borne diseases [3]. Consequently, recent practices involving excessive straw return to the soil have led to the accumulation of *F. graminearum* in wheat fields, triggering the emergence of highly pathogenic and virulent strains, exacerbating widespread outbreaks of FHB, and causing high levels of DON contamination. Chemical pesticides and host wheat resistance breeding currently stand as the primary methods for FHB control. However, chemical pesticide residues pose risks, leading to adverse effects on humans and animals. Moreover, these residues contribute to soil and groundwater pollution, triggering significant environmental and ecological problems [4]. Furthermore, prolonged dependence on a single chemical pesticide can lead to severe resistance in field strains. Host wheat resistance breeding remains the most cost-efficient and eco-friendly strategy for FHB control. Yet, the limited deployment of resistant varieties stems from the scarcity of stable resistance sources possessing desirable yield and yield-related morphological traits. This scarcity persists in cultivated wheat with a narrow genetic base, hampering trait improvement [5]. 

Nowadays, biocontrol via plant growth-promoting bacteria plays a vital role in managing plant diseases [6]. Endophytic bacteria, capable of inhabiting plant tissues internally and establishing a mutual symbiosis relationship, have emerged as a novel source of biochemical pesticides to combat phytopathogens [7]. Also, they can directly inhibit plant pathogens by producing antibiotics or indirectly by inducing systemic resistance in the plant [8]. For example, *Bacillus velezensis* BY6 is an endophytic bacterium that has a good antibacterial effect, simultaneously activating induced systemic resistance and systemic acquired resistance in pathogenic fungus *Alternaria alternate* in *Populus davidiana* x *P. alba* var. *pyramidalis* (PdPap) and promotes the growth of Pdpap seedlings [9]. Similarly, *B. velezensis* SDTB038 is a highly efficient endophytic bacterium that could colonize the tomato plant and produce indoleacetic acid to promote tomato plant growth, which has a better biocontrol effect on *Fusarium* crown and root rot of tomato [10]. Ibrahim et al. documented that the endophytic bacterium *Pseudomonas poae* strain exhibited multifarious antifungal properties through the production of hydrolytic enzymes, siderophores, and lipopeptides, showing potent antifungal activity against *F. graminearum* PH-1 when tested under in vitro and greenhouse conditions [11].

Endophytic bacteria employ several mechanisms in controlling plant diseases, among which the main biocontrol mechanisms include competition, antagonism, and induction of systemic resistance in plants. Endophytic bacteria with biocontrol function compete for nutrients with pathogens and preferentially occupy spatial sites, thereby inhibiting the growth and reproduction of plant pathogens and blocking or interfering with the infection of plant pathogens [10]. At the same time, endophytic bacteria can produce secondary metabolites with antibacterial activity, such as enzymes disrupting fungal cell walls and compounds with fungicidal activity, which can be used as biological agents to promote plant growth and prevent disease [12]. In addition, endophytic bacteria can induce defense enzyme activity and expression of disease-resistance-related genes in host plants, which can induce systemic resistance and increase plant resistance to diseases. Among endophytic bacteria, *Bacillus* sp. has been extensively studied for the biocontrol of fungal phytopathogens [13]. For instance, *B. pumilus* MCB-7 secretes chitinase to dissolve the cell wall of pathogenic fungi [14]. Similarly, *B. subtilis* CB2, isolated from wheat seeds, produces protease, amylase, pectinase, and cellulase, which facilitate bacterial entry into host plants, degrade cell walls, and alter fungal hyphae structures [15]. Therefore, the application of endophytic bacteria holds significant value in the prevention and biocontrol of FHB in wheat fields.

The current work aimed to isolate and fully identify novel bacterial endophyte(s) from wheat seeds to be used as a safe biocontrol agent against *F. graminearum* and the associated mycotoxins in wheat crops. Oxford Nanopore Technology (ONT) single-molecule real-time electrical signal sequencing was used to perform complete genome sequencing and annotation to study the genetic basis and molecular mechanisms underlying the biocontrol effect. The novel isolated strain was tested in vitro and under field trial conditions to confirm the observed biocontrol effect against *F. graminearum* pathogenicity. Quantification of DON and its acetylated form, 15-acetyldeoxynivalenol (15ADON), in wheat, was achieved via liquid chromatography and tandem mass spectrometry (LC-MS/MS). Finally, a plant pathogenicity test and different biosafety assessments were conducted to ensure its safety. This is the first report showing a biocontrol effect of *Pseudescherichia* sp. a strain against phytopathogens.

## 2. Results

### 2.1. Isolation and Identification of Endophytic Bacteria to Control F. graminearum Pathogenicity and DON Production 

**Figure 1:**
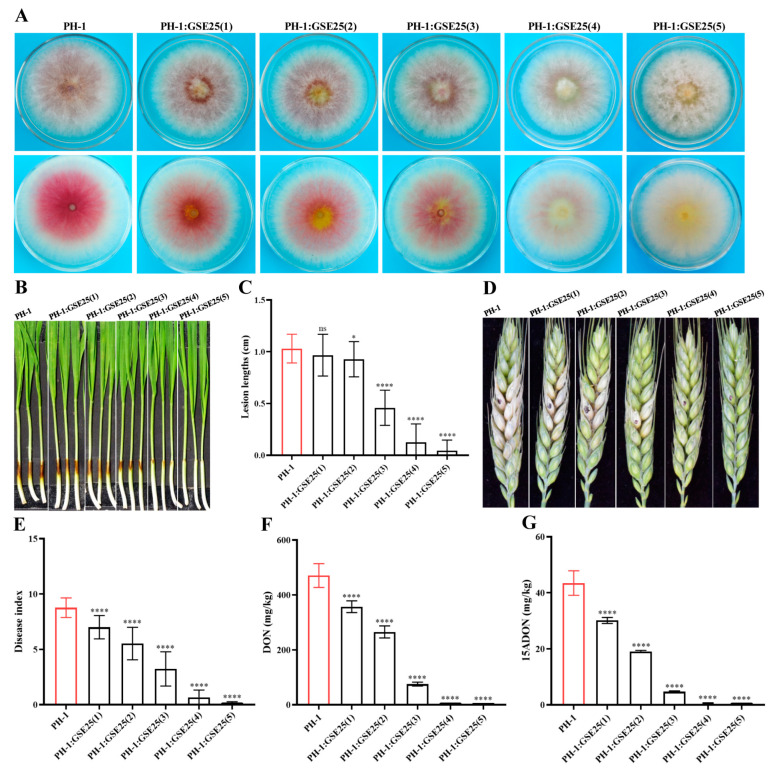
Inhibition of *F. graminearum* PH-1 growth and pathogenicity by *Pseudescherichia* sp. GSE25 strain after multiple interactions. (**A**) *F. graminearum* PH-1 colony morphology. (**B**) Disease symptoms in fresh wheat coleoptile wounds. (**C**) Lesion lengths of the coleoptile. (**D**) Disease symptoms in wheat heads. (**E**) Disease index in wheat heads. (**F**,**G**) Reduction in DON and 15ADON levels in wheat head samples. PH-1:GSE25(1) to (5) represent *F. graminearum* PH-1 after it was purified 1 to 5 times, respectively. Statistical significance compared to *F. graminearum* PH-1 (control treatment) was determined through one-way analysis of variance (ANOVA) (ns: no significance; * *p* < 0.005; **** *p* < 0.0001). Data are expressed as ± standard deviation (SD).

Forty-seven single colonies grown on Luria-Bertani (LB) agar plates were isolated from healthy wheat seeds (*Triticum aestivum* L.) of spring wheat lines Fielder. Based on colony morphology and 16S rRNA sequence identification, a total of 20 isolated strains were grouped into four different genera, including *Pseudescherichia*, *Enterobacter*, *Klebsiella*, and *Yokenella*, with the most abundant genera being *Pseudescherichia* (Table 1). Through multiple interactions with *F. graminearum* PH-1, one isolated strain, namely GSE25, showed a significant reduction in *F. graminearum* pathogenicity and DON production during the initial testing, and it was selected for further study. After multiple interactions with GSE25, phenotypic tests showed that colony growth and hyphal length of *F. graminearum* PH-1 did not change. However, with increasing interaction times, the pigment produced by *F. graminearum* PH-1 became lighter, from red to light yellow (Figure 1A). Notably, by measuring the dark-brown lesion length of wheat coleoptiles, the relative pathogenicity of *F. graminearum* PH-1 was reduced at a rate of 55.5% after the second interaction, while it was 87.9% after the third interaction. A complete loss in *F. graminearum* PH-1 pathogenicity was observed after the fourth and fifth interaction with GSE25 (Figure 1B,C). Additionally, GSE25 demonstrated attenuated pathogenicity of interacted *F. graminearum* PH-1 on flowering wheat heads, compared to the wild-type *F. graminearum* PH-1. The disease index of wheat spikelets during the second and third interactions was reduced to 40.0% and 63.0%, respectively, while no symptoms were observed after the fourth and fifth interactions (Figure 1D,E).

It was speculated that the reduced pathogenicity of *F. graminearum* PH-1 after multiple interactions with GSE25 would affect DON production. Quantifying DON concentrations in the point-of-inoculation wheat spikelet showed a dramatic reduction after two weeks of incubation (Figure 1F). After the first interaction with GSE25, there was a reduction in DON concentration of 24.0% compared with the detected concentrations of DON of the wild-type *F. graminearum* PH-1. After the second and third interactions, a further reduction in DON concentrations was detected of 43.7% and 84.0%, respectively. For the fourth and fifth interactions, almost no DON was detected (a proximately 100% reduction). Furthermore, a significant reduction trend in 15ADON concentrations was the same trend as recorded for DON (Figure 1G). These results demonstrate that strain GSE25 can reduce *F. graminearum* PH-1 pathogenicity and DON production after multiple interactions.

### 2.2. Biosafety Properties, Plant Pathogenicity Test, and Growth-Promoting Ability of Strain GSE25 

**Figure 2:**
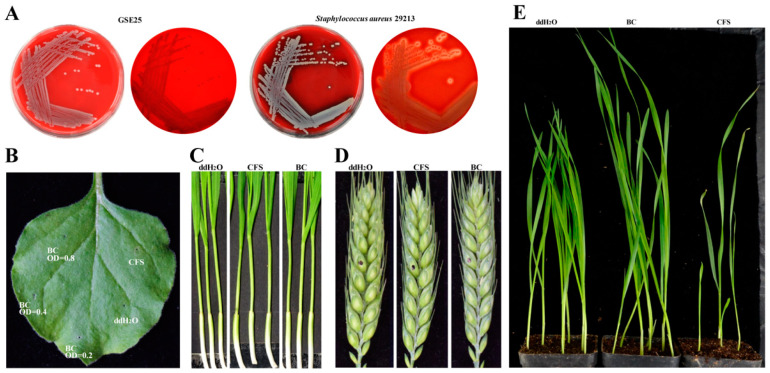
Biosafety evaluation and plant growth-promoting ability assays of *Pseudescherichia* sp. GSE25 strain. (**A**) The hemolytic activity of GSE25 was performed on sheep blood agar plates, and *S. aureus* ATCC 29213 was used as a positive control. (**B**–**D**) Plant pathogenicity test of GSE25 strain. ddH_2_O: double distilled water; CFS: cell-free supernatant of the culture medium of GSE25 strain was filtered three times through 0.22 µm filters; BC: bacteria cells of GSE25 strain washed three times with sterile water. (**E**) Plant growth-promoting effect of GSE25 strain.

Morphological observation showed that GSE25 belonged to gram-negative bacteria, and the colonies were ivory-white, hyaline, and shiny on the LB agar plates (Figure S1). The strain GSE25 did not produce rings of hemolysis around the growth zones on the blood agar plates compared with a strain of *Staphylococcus aureus* 29213 (Figure 2A). To identify the presence of enterotoxins and exotoxins, STa (enterotoxin A, encoded by *estA*), STb (heat-stable enterotoxin B, encoded by *estB*), LT (heat-labile enterotoxin, encoded by *estAB*), and hla (exotoxin Alpha-hemolysin factor, encoded by *hla*) were examined, and the results showed that GSE25 did not carry *STa*, *STb*, *LT* and *hla* genes. To further confirm the plant pathogenic potential of GSE25, the pathogenicity of GSE25 cells (BC), and cell-free supernatant (CFS) were evaluated in wheat coleoptiles, flowering wheat heads, and the leaves of *Nicotiana benthamiana*. Notably, BC and CFS did not cause any disease in wheat (Figure 2B–D). The germination test assessed the plant growth-promoting effects of GSE25. In co-inoculation with BC, the leaf length of seed germination percentage increased by 30.3% compared to the control (Figure S2), while the root length was not significantly increased (Figure 2E). Therefore, the results showed that GSE25 can promote plant growth and seed germination with no pathogenic effect on the tested plants.

### 2.3. Whole Genome Sequence and Phylogenetic Relationship of Strain GSE25

**Figure 3:**
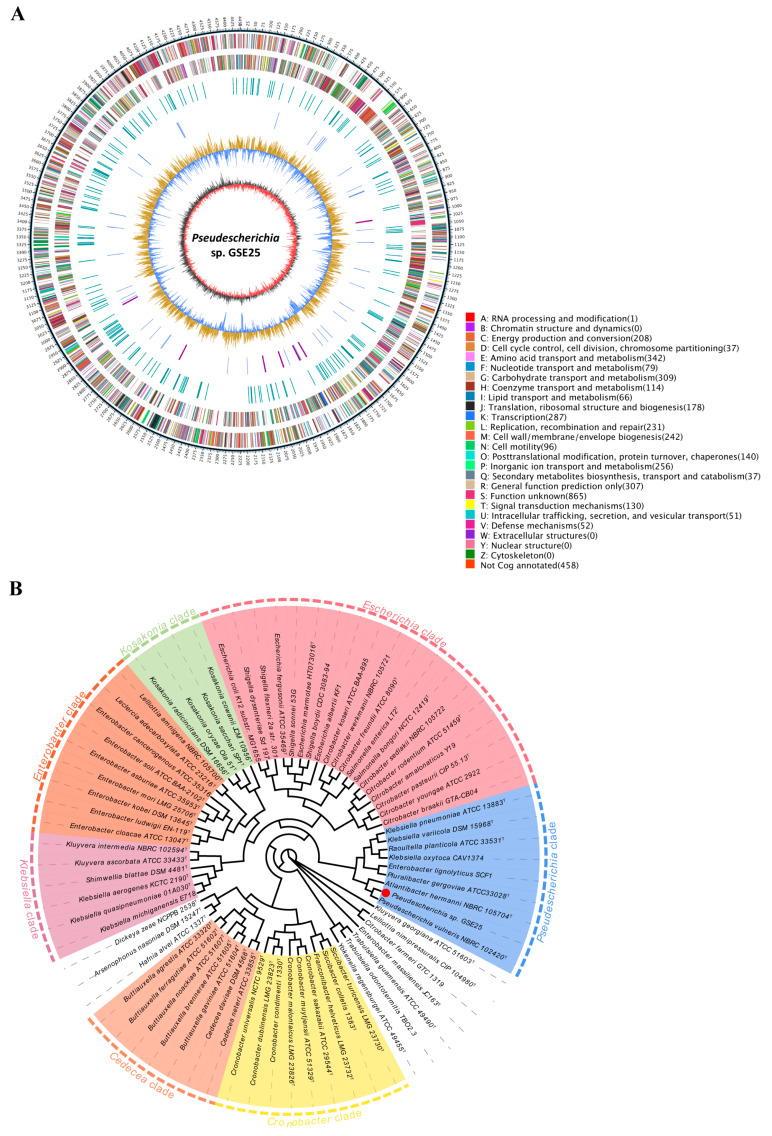
Whole genome map of the *Pseudescherichia* sp. GSE25 strain and its phylogenetic tree together with other 72 *Enterobacteriaceae* species from NCBI database based on genome sequence comparisons. (**A**) The distribution of circles from outwards to inwards are: ring 1 for genome size, each scale is 5 kb (black line); ring 2-3 for COG functional classifications of protein-coding genes on the forward strand and reverse strand, respectively, and different colors represent different COG functional classifications; ring 4 for repeated sequence; ring 5 for the distribution of tRNA (blue) and rRNA (purple); ring 6 for GC content; and ring 7 for GC skew. (**B**) The tree is constructed using the neighbor-joining method with 1000 bootstrap replications. *Pseudescherichia* sp. GSE25 strain is indicated by a red color dot.

To unravel the underlying molecular mechanisms of GSE25 in reducing *F. graminearum* pathogenicity and DON production, the genomic DNA was sequenced, which generated a total of 287,299 clean reads (average read length, 12,475 bp), with an N50 length of 18,768 bp (Table 2). The complete genome of GSE25 comprises a circular 4,761,311 bp chromosome and exhibits a G + C content of 55.6%, with 4486 coding genes, 22 rRNA genes, and 85 tRNA genes (Figure 3A). The genome sequence has been submitted to the SRA under BioSample accession number SAM37224671 and BioProject accession number PRJNA1011830. A total of 4454 unigenes (99.9%) were matched to the NCBI non-redundant (Nr) protein database, which covers about 68.3% of the *Pseudescherichia vulneris* reference genome (Table S1, Figure S3A). Using the evolutionary genealogy of genes: Non-supervised Orthologous Cluster of Orthologous (eggNOG) database on NCBI, 4028 protein-coding genes were matched with 21 different functional classifications in the eggNOG database, which accounts for 89.8% of the entire genome (Figure S3B, Table S1). A total of 2778 protein-coding genes were annotated to three known functional classifications of cellular processes and signaling, information storage and processing, and metabolic processes, of which the metabolic-process-related genes were the most abundant (49.4% of the protein-coding genes). Carbohydrate transport and metabolism and amino acid transport and metabolism accounted for the highest proportion, 25.7% and 24.8% of the total predicted proteins, respectively. In addition, a functional analysis of the gene ontology (GO) database revealed 3322 genes of GSE25 annotated into three categories: biological pathways, cytological components, and molecular functions, with a significant number of genes (1970) related to GO involved in molecular functions (59.3%), and catalytic activity and binding genes accounted for the highest proportion (Figure S3C). The proportion of genes annotated using cytological components (851) was 25.6%. The number of genes annotated using the biological pathway (501) was the lowest, accounting for 15.0%, and metabolic process and single-organism process genes accounted for the highest proportion. Also, the Kyoto Encyclopedia of Genes and Genomes (KEGG) enrichment analysis elucidated the significantly enriched biochemical pathway of annotated genes (2784) in GSE25 (Table S1). The most annotated genes were involved in metabolism, followed by genetic information processing, environmental information processing, and cellular processes (Figure S3D). The major pathways triggered by annotated genes include ABC transporters, biosynthesis of amino acids, a two-component system, and carbon metabolism.

To investigate the phylogenetic inter-relationship of strain GSE25 within the *Enterobacteriaceae* family, the phylogenetic tree in this study contains information for 72 genome-sequenced *Enterobacteriaceae* species from 25 validly published genera within this family, and the tree is presented in Figure 3B. According to the phylogenetic tree, *Enterobacteriaceae* species are classified into seven strongly supported clades, which are labeled as the *Escherichia* clade, *Kosakonia* clade, *Enterobacter* clade, *Klebsiella* clade, *Cronobacter* clade, *Cedecea* clade, and the *Pseudescherichia* clade. The clade relationship of GSE25 belongs to the genera *Pseudescherichia*. Therefore, GSE25 was designated as *Pseudescherichia* sp. GSE25. This strain was deposited at the China General Microbiological Culture Collection Center (CGMCC), with strain accession number CGMCC No. 26158.

### 2.4. DNA-seq and RNA-seq Analyses of Inhibitory Effects of Strain GSE25 on F. graminearum PH-1

**Figure 4:**
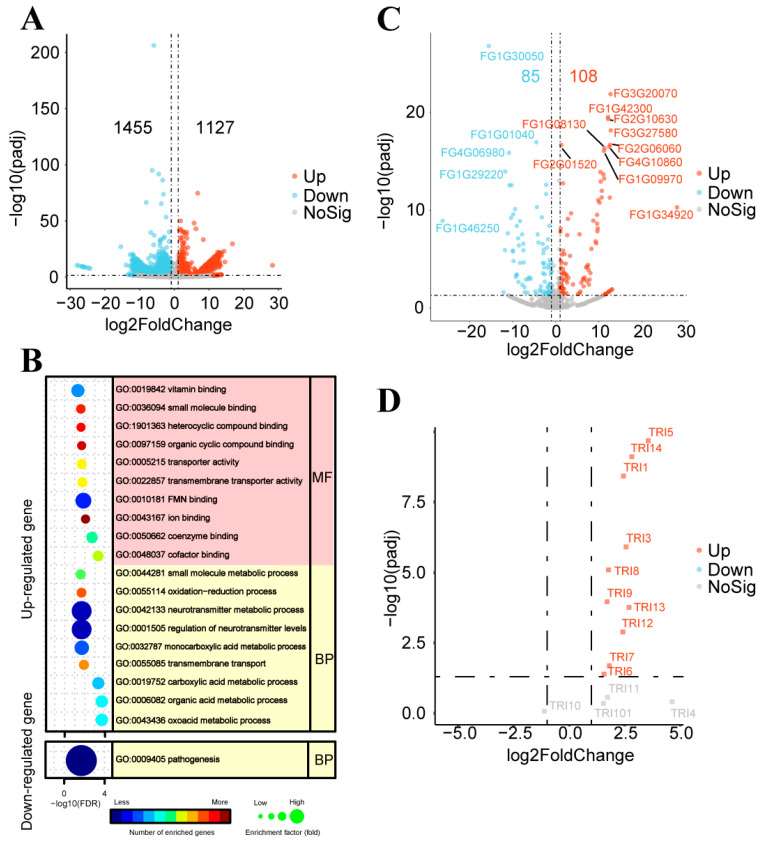
Volcano map of DEGs and GO functional classification of *F. graminearum* PH-1 after interaction with *Pseudescherichia* sp. GSE25 strain via RNA-Seq analysis. (**A**) Volcano plot of 2582 differential expressed transcripts. (**B**) Dot plot of GO enrichment analysis of DEGs. (**C**) Volcano plot of DEGs in reported functional genes of *F. graminearum*. The top dots are named gene ID of *F. graminearum* PH-1 (YL1 version). (**D**) Volcano plot of *TRI* gene clusters in *F. graminearum*.

Single nucleotide polymorphism (SNP) in *F. graminearum* PH-1 after five consecutive interactions with strain GSE25 (GSE25:PH-1(5)), which was verified for the gene mutation site by DNA sequencing. The SNP analyses showed mutation sites on the four chromosomes of *F. graminearum* PH-1 interaction with GSE25, all in the non-coding region (Table S2). To further explore the function of GSE25, RNA sequencing was performed on GSE25:PH-1(5) and the wild-type *F. graminearum* PH-1 as a control. Transcriptome analysis revealed that 2582 differentially expressed transcripts were obtained in GSE25:PH-1(5) compared with the wild-type *F. graminearum* PH-1, including 1127 up-regulated and 1455 down-regulated differential expressed transcripts (Figure 4A). Meanwhile, the GO enrichment analysis was identified. The up-regulated differentially expressed genes (DEGs) were mainly involved in biological processes such as regulation of neurotransmitter levels and neurotransmitter metabolic process and molecular functions such as FMN binding and vitamin binding (Figure 4B). On the other hand, the down-regulated DEGs showed that only biological processes involved in pathogenesis were enriched. These results suggested that the interaction between *F. graminearum* PH-1 and GSE25 could reduce the pathogenicity of *F. graminearum* PH-1 by regulating the expression of genes in the pathogenic process. 

To further verify the obtained results, some of the functionally validated genes, previously reported (Table S3) to be crucial in the pathogenesis of *F. graminearum*, were analyzed for gene expression patterns of GSE25:PH-1(5). Interestingly, *FgKP4L-2* (FG1G01040), *TOP1* (FG4G06980), and *FgATF1* (FG1G46250) genes significantly reduced pathogenicity when knocked out in *F. graminearum*, were all present in the down-regulated DEGs (Figure 4C). *TPS1* (FG3G20070), *ROA* (FG2G10630), *AUR1* (FG1G34920), and some transcription factors were found in up-regulated DEGs. Furthermore, to search for trichothecene biosynthesis (*TRI*) gene clusters responsible for DON biosynthesis in *F. graminearum*, 14 *TRI* genes were screened in the DEGs of GSE25:PH-1(5). It was found that the *TRI* genes showed dramatically up-regulated expression, especially the *TRI5*, *TRI14*, and *TRI1* genes (Figure 4D). These results suggest that GSE25 attenuates the pathogenicity and directly affects the *TRI* gene expression in *F. graminearum* PH-1. Raw data from the DNA-seq and RNA-seq experiments generated in this study were deposited in the NCBI under the accession number PRJNA1017085.

### 2.5. In Vitro Effect of Strain GSE25 on F. graminearum

**Figure 5:**
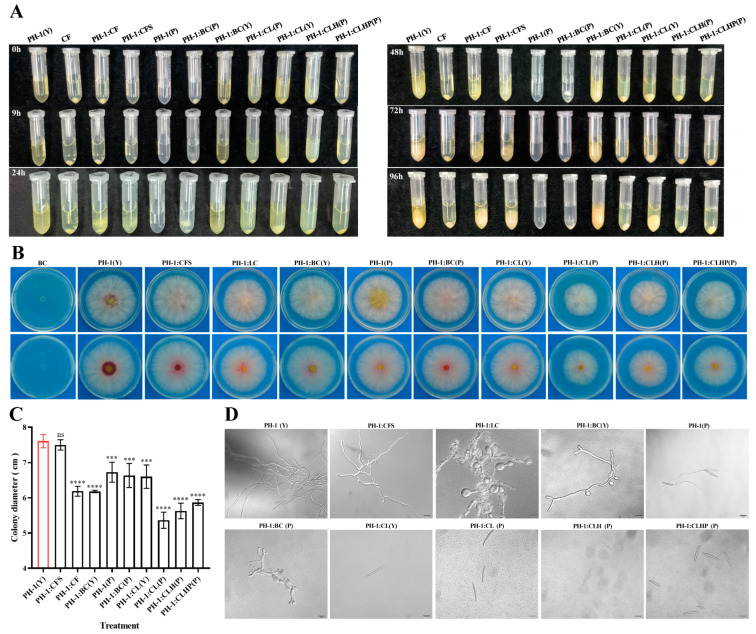
In vitro antifungal activity of *Pseudescherichia* sp. GSE25 strain against *F. graminearum* PH-1 under different treatment conditions. (**A**) The morphological appearance of *F. graminearum* PH-1 growth at different time points (0, 9, 24, 48, 72, and 96 h). PH-1(Y): spore suspension in YEPD medium; CF: culture fluid of GSE25; CFS: cell-free supernatant of the culture medium of GSE25; PH-1(P): spore suspension in PBS buffer; BC(P): bacteria cells of GSE25 (BC) in PBS buffer; BC(Y): BC in YEPD medium; CL(P): BC ultrasonically broken until the cell lysate (CL) in PBS buffer; CL(Y): CL in YEPD medium; CLH(P): CL in PBS buffer heat at 60 °C for 15 min; CLHP(P): CL in PBS buffer with 200 µL protease K (100 μg/mL) heat at 60 °C for 15 min. (**B**,**C**) Colony morphology and growth rate of *F. graminearum* PH-1 with different treatments on PDA agar grown at 25 °C for three days. (**D**) Hyphal and spore morphologies of *F. graminearum* PH-1 under light microscope after different treatments for 24 h. Scale bar = 20 μm. Statistical significance compared to *F. graminearum* PH-1 (control treatment) was determined through one-way analysis of variance (ANOVA) (ns: no significance; *** *p* < 0.001; **** *p* < 0.0001). Data are expressed as ± standard deviation (SD).

Different assays were established to assess the antifungal activities. *F. graminearum* PH-1 was co-inoculated with different liquid culture treatments of GSE25 in yeast extract peptone dextrose medium (YEPD) medium or PBS buffers. The liquid assay allowed a time-dependent monitoring of the conidia response to different culture liquid treatments (Figure 5A). After 24 h, hyphae growth was observed in the liquid of *F. graminearum* PH-1(Y), culture fluid of GSE25 (CF), CFS, and BC(Y), and the quantity of hyphae increased with a longer culture time. However, hyphae formation was not observed in the liquid of cell lysate CL(P), CLH(P), CLHP(P) and CL(Y). The liquid assay suggests that CL inhibited spore germination into hyphae. A growth assay on potato dextrose agar (PDA) agar plates was conducted for 9 h (Figure 5B). Except for the CFS, a more than 25% reduction in colony growth of *F. graminearum* PH-1 was observed for CL(P), CLH(P), and CLHP(P), and about 15% of the reduction was observed for CF, BC(Y) BC(P), and CL(Y) compared to the un-inoculated controls (Figure 5C). It was worth noting that the CL affected the colony growth of *F. graminearum* PH-1, while CFS had no effect.

For spore germination, microscopic observation indicated that *F. graminearum* PH-1 hyphae in the CF were significantly altered (abnormal, swollen, nearly spherical, and damaged, with broken and destructed cell walls), and sporulation was inhibited (Figure 5D). Results showed that *F. graminearum* PH-1 hyphae in BC(P) were abnormal and swollen, but the branches of hyphae were partially inflated in BC(Y), and sporulation was inhibited in both BC(P) and BC(Y). Remarkably, spore germination was completely inhibited in CL(P), CLH(P), and CLHP(P), while spore germination occurred in a single-ended manner in PBS buffer, and a germ tube appeared at one end of the spore in CL(Y), but it was very short. These observations suggest that the CL can effectively inhibit the spore germination of *F. graminearum* PH-1, CF, and BC, leading to abnormal hyphae and nearly spherical swelling. 

**Figure 6:**
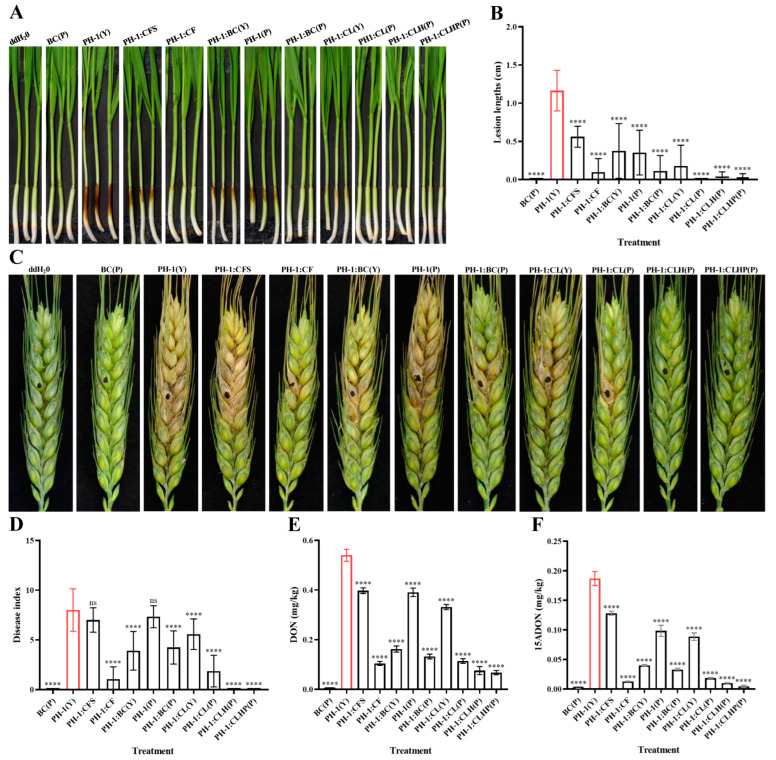
Biocontrol effect of *Pseudescherichia* sp. GSE25 strain against *F. graminearum* PH-1 and the associated mycotoxins in wheat coleoptiles and heads. (**A**) Disease symptoms in fresh wheat coleoptile wounds. ddH_2_O: double distilled water; BC(P): bacteria cells of GSE25 (BC) in PBS buffer; PH-1(Y): spore suspension of PH-1 in YEPD medium; CFS: cell-free supernatant of the culture medium of GSE25; CF: culture fluid of GSE25; BC(Y): BC in YEPD medium; PH-1(P): spore suspension of PH-1 in PBS buffer; CL(Y): BC ultrasonically broken until the cell lysate (CL) in YEPD medium; CL(P): CL in PBS buffer; CLH(P): CL in PBS buffer; CLHP(P): CL in PBS buffer with 200 µL protease K (100 μg/mL) heat at 60 °C for 15 min. (**B**) Lesion lengths of the coleoptile area. (**C**) Disease symptoms of wheat heads. (**D**): Disease index in different wheat head groups. (**E**,**F**) Effect of strain GSE25 on DON and 15ADON levels. Statistical significance compared to *F. graminearum* PH-1 (control treatment) was determined through one-way analysis of variance (ANOVA) (ns: no significance; **** *p* < 0.0001). Data are expressed as ± standard deviation (SD).

After the primary agar plate and microscopic observation, different culture fluid treatments were tested by measuring wheat coleoptile infection. Results showed that the dark-brown lesion of the wild-type *F. graminearum* PH-1 almost covered the coleoptile area (about 1.2 cm) as a positive control (Figure 6A). At the same time, those treated with BC and CL, including CF, BC(P), CL(P), CLH(P), and CLHP(P), effectively reduced the pathogenicity of *F. graminearum* PH-1. Remarkably, the relative pathogenicity of the treated samples with CFS, BC(Y), and PH-1(P) was decreased from about 53.7% to 69.8%, and CL(Y) demonstrated attenuated virulence, as the light-brown lesion size was about 0.2 cm (Figure 6B). For flowering wheat heads, the infected wheat spikelets treated with BC and CL were dramatically reduced (Figure 6C,D). A number of diseased wheat spikelets were about 8 grains in positive control and treated with CFS after two weeks post-inoculation. The obtained data showed that no diseased wheat heads were found in the CLH and CLHP, and the disease index of CF and CL(P) decreased by 86.9% and 76.8%, respectively. The treated conditions with BC(Y), BC(P), and CL(Y) effectively reduced pathogenicity by 51.3%, 47.0%, and 30.3%, respectively. The obtained data suggest that cell lysate of strain GSE25 significantly reduces the pathogenicity of *F. graminearum*, especially after heating and protease K treatment.

To further determine whether strain GSE25 plays a crucial role in DON production during interaction with *F. graminearum*, DON levels were measured in different culture fluid treatments of GSE25 on wheat spikelet. Figure 6E showed that strain GSE25 significantly reduced DON levels in treated groups with CF, CL(P), CLH, and CLHP by 80.7%, 78.9%, 86.0%, and 87.5%, respectively. Furthermore, DON levels of BC(Y) and BC(P) were decreased by 69.9% and 75.5%, respectively. Meanwhile, DON concentrations decreased by 38.6%, 26.4%, and 27.6% in the treatment of CL(Y), CFS, and PH-1(P), respectively. Consistently, 15ADON levels in different culture fluid treatments were significantly reduced in the infected wheat spikelet. These data indicate that the treatment of CF, CL(P), CLH(P), and CLHP(P) resulted in a 90% to 97% reduction in 15ADON levels compared to the wild-type PH-1, the treatment of BC(Y) and BC(P) resulted in a 78% to 82% reduction, and the treatment of CFS, PH-1(P), and CL(Y) resulted in a 31% to 52% reduction (Figure 6F). The current findings suggest the potential of strain CSE25 in reducing DON accumulation in wheat infected with *F. graminearum* PH-1.

### 2.6. Biocontrol Effect of Strain GSE25 on FHB in Field Trials

**Figure 7:**
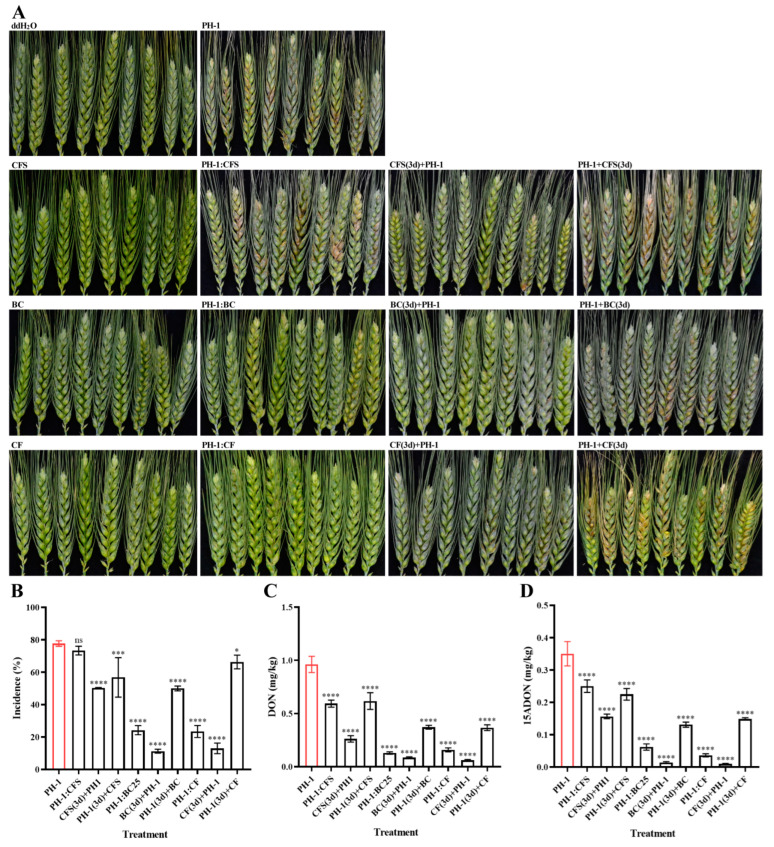
Biocontrol effect of *Pseudescherichia* sp. GSE25 strain against FHB disease observed during 2022–2023 field trials using wheat cultivars. (**A**) Disease symptoms in wheat heads inoculated with *F. graminearum* PH-1 with different treatment groups. CFS: cell-free supernatant of the culture medium of GSE25; BC: bacteria cells of GSE25; CF: culture fluid of GSE25; PH-1:CFS, PH-1:BC, and PH-1:CF: a co-spraying with spore suspension of *F. graminearum* PH-1 with CFS, BC, and CF, respectively, mixed in a 1:1 ratio; CFS(3d) + PH-1, BC(3d) + PH-1, CF(3d) + PH-1: sprayed with CFS, BC, and CF for three days, respectively, and then sprayed with spore suspension of *F. graminearum* PH-1; PH-1 + CFS(3d), PH-1 + BC(3d), PH-1 + CF (3d): sprayed with spore suspension of *F. graminearum* PH-1 for three days, and then sprayed with CFS, BC, and CF, respectively. (**B**) Disease incidence (%) of FHB disease in different treatment groups and the control. (**C**,**D**) Reduction in DON and 15ADON levels in wheat heads. Statistical significance compared to *F. graminearum* PH-1 (control treatment) was determined through one-way analysis of variance (ANOVA) (ns: no significance; * *p* < 0.005; *** *p* < 0.001; **** *p* < 0.0001). Data are expressed as ± standard deviation (SD).

Flowering wheat heads of spring wheat line Fielder were used to determine the antifungal activity of strain GSE25 in the plants. Comparisons were made among flowering wheat heads sprayed with CFS, BC, and CF treatment groups. Spore suspension of the wild-type *F. graminearum* PH-1 was a positive control, while sterile water was a negative control. Results showed a significant difference between the treatments and the negative control in all parameters. In the positive control, infected wheat heads could be observed in almost every wheat plant, and the incidence of the wild-type *F. graminearum* PH-1 was 77.7%, while no disease symptoms were detected in the negative controls (Figure 7A,B). In the co-spraying treatment group, the incidence of CFS, BC, and CF on *F. graminearum* was observed at 73.4%, 24.2%, and 23.4%, respectively. After spraying the spore suspension of *F. graminearum* PH-1 for three days, the flowering wheat heads were sprayed with different treatments, and the incidence of CFS, BC, and CF on *F. graminearum* was observed at 56.8%, 50.0%, and 66.3%, respectively. Remarkably, when the flowering wheat heads were sprayed with different treatments for three days and then sprayed with the spore suspension of PH-1, the incidence of CFS, BC, and CF on *F. graminearum* were observed 50.2%, 11.3%, and 13.0%, respectively. The obtained data indicate that spraying strain GSE25 in wheat fields can effectively control the infection of *F. graminearum*, especially before the occurrence of the disease.

To further investigate the effect of strain GSE25 on *F. graminearum* infection and DON accumulation in wheat. DON concentrations were determined via LC-MS/MS. Wheat heads sprayed only with the wild-type PH-1 revealed the highest level of DON and 15ADON, and the average concentrations were 961.7 µg/kg and 350.4 µg/kg, respectively (Figure 7C,D). In the co-spraying treatment group, DON and 15ADON levels in CFS treatment decreased the lowest, only 38.3% and 28.6% of the positive control group, respectively, and the detected concentrations of DON and 15ADON in BC and CF treatment groups significantly decreased, with a reduction rate from 82% to 89%. After spraying the spore suspension of *F. graminearum* PH-1 for three days, wheat heads were sprayed with different treatments, and the concentrations of DON and 15ADON in CFS, BC, and CF treatment groups were decreased by 35.8%, 61.2%, and 61.8% and 35.9%, 62.6%, and 57.6%, respectively. When wheat heads were sprayed with different treatments for three days and then sprayed with the spore suspension of PH-1, except for the CFS treatment group, the DON and 15ADON concentrations were reduced by more than 91% in both BC and CF treatment groups. Altogether, the obtained data imply that spraying the strain GSE25 in the field can effectively reduce the accumulation of DON in wheat grains. Our data further suggest that strain GSE25 can be used as a bacteriological agent in fields for the biocontrol of FHB.

## 3. Discussion

FHB, caused by the plant pathogen *F. graminearum*, has become one of the most critical contributors to wheat yield losses and mycotoxin contamination. In the flowering period of wheat, moist weather is conducive to the spread of FHB. Currently, fungicides are mainly used to prevent and control FHB in the wheat flowering period. However, the resistance of *F. graminearum* to chemical pesticides increases with excessive use and even results in ineffective fungicides [11]. Hence, there is an urgent need to isolate and identify effective endophytic microbes from wheat fields to be used as an alternative to synthetic chemicals. The endophytic microbes are naturally harbored in many plant seeds and play crucial roles in plant health and yield, thereby protecting against plant pathogens [16]. Previously, six bacterial endophytes were isolated from the seeds of a commercial wheat cultivar widely sown in Argentina, assigned to the genera of *Paenibacillus*, *Bacillus*, and *Pantoea*. The inoculation with one of *Paenibacillus* isolates combined with *Pantoea* isolate could display greater biocontrol to *F. graminearum* and increase the chlorophyll content of barley seedlings [17]. Metabolites produced by the endophytic *B. mojavensis* PS17, which was isolated from surface-sterilized wheat seeds of the variety Sadokat (Tatdjik Republic), inhibited the growth of *F. graminearum* and showed potential as a biocontrol agent for agricultural use [18]. Recently, a *B. subtilis* strain CB2 isolated from eight cultivars of wheat surface-sterilized seeds in Iran could produce surfactins and iturins that revealed activity in the biocontrol of *F. graminearum* [15]. In this study, 20 endophytic bacterial strains were isolated and identified from healthy wheat seeds of spring wheat line Fielder and characterized into four different genera, with the most abundant genera being *Pseudescherichia* (Table 1). These results are consistent with the previous findings, which showed that seeds have fewer microbial resources than other wheat tissues. *Bacillus* species are one of the most convincing biocontrol mechanisms with broad-spectrum antifungal activity and are the dominant endophytic bacteria isolated from wheat seeds [18]. However, this was not the case in the current study, possibly due to the geographical isolation environment or the difference in wheat cultivars.

As not all endophytic bacteria of wheat seeds can inhibit fungal growth, the antifungal activity of *Pseudescherichia* species was verified during the interaction with *F. graminearum*. In this study, the strain GSE25 demonstrated significant inhibition of *F. graminearum* growth and reduction of DON levels after multiple interactions (Figure 1). The strain GSE25 is a gram-negative bacterium and phenotypically challenging to distinguish from the family *Enterobacteriaceae* and other *Pseudescherichia vulneris* species based on 16S rRNA sequence similarity. It has been reported that *P. vulneris* was detected in several clinical isolates and had the potential to cause diarrheal illness by examining the genes for enterotoxins and exotoxin [19,20,21]. To ensure the availability and biosafety of GSE25 on crops, we tested the virulence factors and hemolysis of GSE25. Results show that GSE25 did not contain enterotoxins, exotoxin, and hemolysin genes and did not produce the rings of hemolysis around the growth zones on the blood agar plates (Figure 2A). This is consistent with the recent findings in which a strain belonging to the species *P. vulneris* was isolated from the tomato stems, indicating that it is incapable of causing diarrhea [21]. Additionally, some endophytes can effectively promote the growth of plants but are influenced by the local environment and the genotype of the host, and the mechanisms of plant growth-promoting effect of endophytes remains elusive [22]. To explore the growth-promoting effect of the strain GSE25, BC and CFS were used to irrigate wheat seeds, and seed germination and plant growth were observed. In this study, seed germination and the phenotype of the plants after the irrigation of BC were better than those of the CFS treatment and control treatment, including more leaves and a larger leaf area (Figure 2E). Nevertheless, seeds treated with CFS showed inhibited germination or slow plant growth even when germinated. This indicates that some components of the LB medium or some species produced by GSE25 in the medium may be unfavorable to seed germination and plant growth. As previously reported, GSE25 may modulate plant growth by providing or regulating various plant hormones or indirectly promoting plant growth by limiting or preventing plant damage that might otherwise be caused by various pathogens [22]. Since GSE25 has a good growth promotion effect on plants, its specific mechanism will be further understood in the future. In this study, BC can be considered as an excellent material for subsequent biocontrol of FHB. Also, in the current work, it was shown that the GSE25 strain is a non-opportunistic pathogen and safe for animals and humans, giving it a chance to be tested in a large-scale field experiment, which will advance our knowledge of the use of GSE25 for biocontrol of plant pathogens.

*Pseudescherichia vulneris* was initially classified as *Escherichia vulneris* within the genus *Escherichia* and was later divided into novel genera viz. *Pseudescherichia* gen. nov. due to its genetic variability [21,23]. Meanwhile, little was reported about *P. vulneris*, which is only isolated from tomato stems, activated sludge, and soil samples of abandoned mine areas [21,24,25]. To understand the interrelationships between the GSE25 and the *Enterobacteriaceae* species, we sequenced the complete genome of GSE25 and constructed a phylogenetic tree for 72 genome-sequenced *Enterobacteriaceae* species from 25 validly published genera within this family (Figure 3). Genome-guided assembly demonstrated that the genomic reads of GSE25 matched only to the genome of *P. vulneris* with a coverage rate of 68.3% (Figure S3). Thus, the isolated strain was phylogenetically distant from known strains and referred to as *Pseudescherichia* sp. GSE25 belongs to the *Proteobacteria phylum*, *Gammaproteobacteria* class, *Enterobacteriales* order, *Enterobacteriaceae* family, and *Pseudescherichia* genus. To our knowledge, this is the first report of *Pseudescherichia* species found in the endophytic population of wheat seeds.

Endophyte-fungal interactions in plants widely impact crop productivity and range from highly beneficial to detrimental [26]. To gain insight into the role of endophytes in protecting wheat plants, a sequence analysis of the interaction between endophyte GSE25 and fungus *F. graminearum* was conducted. The DNA-seq analysis indicated that GSE25 did not affect the genetic traits of *F. graminearum* due to all the mutation sites occurring in the non-coding region (Table S1). Transcriptome analysis revealed that the down-regulated DEGs were only impacted by the pathogenesis of biological processes. From these observations, it becomes clear that GSE25 can directly affect gene expression in *F. graminearum*, possibly contributing to reduced pathogenicity in wheat plants. These are most likely endophytic fungus *Serendipita vermifera,* through reduced expression of effector genes to reduced pathogenicity of *Bipolaris sorokiniana* in barley roots, shows a direct biocontrol effect already outside of the root, hence its designation as a ‘gatekeeper’ [27]. Furthermore, RNA-seq analyses highlighted the 193 DEGs whose function were reported in *F. graminearum* (Figure 4C). Interestingly, *FgKP4L-2* (FG1G01040), *TOP1* (FG4G06980), and transcription factor gene *FgATF1* (FG1G46250) were found in down-regulated DEGs with a significant expression. The expression of the *FgKP4-2* gene was previously shown to be associated with fungal pathogenesis in FHB disease [28]. The topoisomerase I protein (TOP1) was identified as a potential virulence factor of *F. graminearum* from a forward genetics experiment and may be a suitable target for chemical control due to the importance in pathogenicity of *Fusarium* sp. on wheat ears [29]. In addition, deletion of the *FgATF1* gene from *F. graminearum* showed pleiotropic phenotypes, including reduced growth rate, virulence, and DON production in infested wheat kernels [30,31]. Notably, it was found that *TPS1* (FG3G20070), *ROA* (FG2G10630), and *AUR1* (FG1G34920) were in up-regulated DEGs. It was indicated that Trehalose 6-phosphate synthase (TPS1) was dispensable for the fungal development and virulence in *F. graminearum* [32]. Deletion of the *ROA* gene from *F. graminearum* resulted in an abnormal size and shape of asci and ascospores but did not affect vegetative growth [33]. Besides, AUR1, as the polyketide synthases responsible for the biosynthesis of the red mycelium pigment aurofusarin in *F. graminearum,* was expressed during fruiting body development, and *AUR1* mutant failed to produce aurofusarin [34,35]. The expression pattern of the *AUR1* gene may be related to the light-colored pigment produced by *F. graminearum* on the PDA plate after multiple interactions with strain GSE25. DON is a vital virulence factor that contributes to the spread of *F. graminearum* infection [36]. Therefore, by analyzing the RNA-seq data, most of the *TRI* genes appeared in up-regulated DEGs when *F. graminearum* was co-grown with strain GSE25 in the PDA medium (Figure 4D). Under biological stress by GSE25, *TRI* gene expressions seem to be enhanced, although they had minimal expression. According to a previous study, hydrogen peroxide supplementation in the culture medium leads to the expression of *TRI* genes in *F. graminearum* and increased DON production [37]. This finding suggests that these *TRI* genes in *F. graminearum* during interaction with GSE25 have a similar expression pattern as that of oxidative stress induced by hydrogen peroxide.

This study investigated the effect of the bacterial cell, culture fluid, cell-free supernatant, and cell lysate of GSE25 on the development of *F. graminearum*. The growth of *F. graminearum* was simultaneously monitored in the presence and absence of strain GSE25 (Figure 5). Treatment with the strain GSE25 showed consistent antifungal activities against *F. graminearum*. Colony growth of PH-1 was inhibited at a range between 15% and 25%. In agreement with the obtained results from the current study, previous studies have indicated that the endophytic *B. vallismortis* ZZ185 and *P. poae* strain CO can be used as antifungal agents to suppress *F. graminearum* [11,38]. The fungal cell wall is crucial in determining cell shape and protecting fungal cells from external stress [39]. in vitro antifungal experiments revealed that, in co-inoculation treated with CF and BC containing viable cells, the hyphae of *F. graminearum* PH-1 showed abnormal swelling and bulbous structures, which turned it in to “balloons” with different ratios and even had a strongly inhibitory effect on the sporulation (Figure 5D). The viable cells of GSE25 can disrupt the walls and membrane systems of pathogenic *F. graminearum*, impairing its ability to infect plants and produce DON (Figure 6). This might be because endophytic bacteria disrupt the natural morphological structure and interfere with their normal proliferation. Similar effects of many endophytic bacteria have been reported on the cell walls of pathogenic fungi, such as *Streptomyces albus* CINv1, *P. poae* strain CO, and *B. velezensis* L1 [11,40,41].

It is well known that spore germination plays a crucial role in plant colonization and infection of pathogenic fungi [42]. In this study, CL at four different treatments, including CL(Y), CL(P), CLH(P), and CLHP(P), were able to effectively suppress the spore germination and germ tube growth of *F. graminearum*. In the presence of CL, spore germination was completely inhibited, while in the YEPD medium as a control, only slightly short germ tubes grew out (Figure 5D). In addition, it was noted that DON levels in wheat spikelets co-inoculated with *F. graminearum* and BC or CL were significantly reduced to 69% (Figure 6). At the same time, the pathogenicity of wheat coleoptiles and heads were significantly reduced, with no symptoms. Therefore, these results indicated that the intracellular components of GSE25 could effectively inhibit spore germination and significantly reduce the infection risk of *F. graminearum* in wheat plants. In this study, we provide evidence that the viable cells or the cell lysate of GSE25 have antifungal properties. Notably, CFS did not exhibit the above inhibitory effects compared with the control treatment. Therefore, we further provide strong evidence that this antifungal effect is not primarily linked to secondary metabolites but also to other proteinaceous factors that still need to be identified.

FHB can infect wheat plants at different stages, from sowing to heading, which renders the outbreak of FHB in wheat fields more serious [43]. Additionally, the accumulation of DON in wheat grains threats the safety of wheat used for human consumption. Due to the scarcity of resistant varieties, the use of fungicides has become the prevalent approach to control the FHB in farmland. Furthermore, excessive or long-term use of chemical fungicides increases the resistance of *F. graminearum* [11]. For these reasons, the use of endophytic bacteria as a biocontrol agent is a new strategy that has a great potential to control phytopathogens. Beneficial endophyte inhabits the intercellular spaces of plants and establishes a self-reproducing system, infecting the plant tissues throughout the lifecycle without causing obvious lesions [44]. Although there are a considerable number of published reports on bacterial endophytes to provide plant protection against many diseases, many documented biocontrol agents are still stuck at the in vitro lab scale. Therefore, searching for novel and effective biocontrol agents is of a paramount importance. In this study, we isolated the endophytic bacterium GSE25 from wheat seeds, which showed a potent antifungal effect on *F. graminearum*. To further confirm the biocontrol effects of GSE25 on wheat FHB disease, we performed an in-field test on the flowering wheat headings. The actual assessment of the biocontrol effect of the GSE25 on FHB can be better reflected under natural field conditions. The results showed that applying the strain, GSE25 significantly reduced the FHB disease. Here, co-spraying of GSE25 with *F. graminearum* showed inhibitory effects on wheat heads, and the FHB incidence was significantly reduced to more than 60%. In particular, the effect of spraying GSE25 three days before the inoculation of *F. graminearum* was more effective as the FHB incidence was reduced by 85.5% (Figure 7B). This is strong evidence of the biocontrol potential of GSE25 to control FHB disease. In agreement with these results, the endophytic bacteria *B. amyloliquefaciens* stain XS-2, *B. mojavensis* strain RRC 101, and *P. poae* strain CO were able to suppress the disease severity of FHB under greenhouse conditions [11,43,45]. Furthermore, the results showed that DON concentrations in wheat spikelet co-spraying *F. graminearum* with GSE25 were significantly reduced to 86.6% compared to samples sprayed only with *F. graminearum* (Figure 7C). Likewise, the results of 15ADON concentration were reduced to 82.4% (Figure 7D). In the meantime, the DON and 15ADON concentrations in wheat spikelets sprayed with GSE25 three days before the inoculation of *F. graminearum* were reduced significantly to 91.1% and 96.0%, respectively. Previous studies have also explored the endophytic bacteria *B. velezensis* YB-130 and *B. megaterium* BM1, which were isolated from wheat spikes and grains, respectively, and were able to suppress the DON levels by FHB, which were matching our results [36,46].

## 4. Conclusions

FHB is a destructive fungal disease that causes extensive yield and quality losses in wheat. Biocontrol of FHB is considered an alternative disease management strategy, especially to reduce the harm caused by chemical pesticides to humans and the environment. Hence, the use of endophytic microbes is promoted under sustainable and green agriculture concepts. In this study, an endophytic *Pseudescherichia* sp. GSE25 strain from wheat seeds was isolated and identified. The strain GSE25 showed strong antifungal activity against FHB pathogen *F. graminearum* strain PH-1 and DON accumulation in vitro and under field conditions. The biocontrol effect of GSE25 was mainly attributed to the damage of the *F. graminearum* cell membrane, inhibition of spore germination, and suppression of germ tube growth. This resulted in reduced pathogenicity of wheat plants and DON accumulation in wheat grains. Thus, the isolated bacterial endophyte is an excellent alternative candidate to chemical fungicides, and effectively protects wheat from toxigenic fungal infection while promoting plant growth.

## 5. Materials and Methods

### 5.1. Plant, Fungal, and Bacterial Materials

To isolate endophytic bacteria, wheat seeds were collected from healthy wheat plants (spring wheat line Fielder) at the Caoxinzhuang Wheat Research Farm in Yangling, Shaanxi, China. Wheat cultivar Xiaoyan22 and spring wheat line Fielder were planted in Caoxinzhuang Wheat Research Farm during the wheat season (November–July in 2022 and 2023). The flowering wheat heads of cultivar Xiaoyan22 were inoculated for wheat spikelet infection assay, and the flowering wheat heads of spring wheat line Fielder were used to determine the incidence of the FHB. Nine seeds of spring wheat line Fielder were sown in 7 cm × 8 cm plastic plot size with a spacing of 2.5 cm between plants and 2.5 cm between rows in a shaded greenhouse at 25 °C with 90% relative humidity and a 14 h/10 h photoperiod. After nine days of plant growth, fresh wheat coleoptile wounds were used to observe the disease symptoms of *F. graminearum* fungi. *Nicotiana benthamiana* were grown for five weeks in plastic pots (10 cm × 10 cm) to evaluate biosafety of endophytic bacteria. The wild-type *Fusarium graminearum* PH-1 was provided by the State Key Laboratory of Crop Stress Resistance and High-Efficiency Production, Northwest A&F University. The strains *S. aureus* ATCC 29213 and enterotoxigenic *Escherichia coli* ETEC were provided by the College of Food Science and Engineering, Northwest A&F University.

### 5.2. Isolation and Identification of Endophytic Bacteria from Wheat Seeds

Endophytic bacteria were isolated from wheat seeds according to the method of Ibrahim et al. [11] with some modifications. Ten grams of each sterile tissue were separately crushed, mixed with 50 mL of saline water (0.85% NaCl), and subjected to serial dilution up to 10^−3^. From each dilution (three replicates), 100 µL was cultured on LB agar plates at 25 °C for 1−2 days. Microbial colonies were purified by transferring single colonies to new LB agar plates. The isolated strains were stored in 20% (*v*/*v*) glycerol at −80 °C until further use. The isolated endophytic bacteria was identified using the 16S rRNA gene and sequence analysis, according to Ibrahim et al. [11]. Sequences from the 16S rRNA genes were spliced using DnaMan V6 and used for homology search using the Basic Local Alignment Search Tool (BLAST) software 2.13.0 algorithm and comparison with the GenBank nucleotide data bank from NCBI. The predominant strain was selected for subsequent experiments.

### 5.3. Screening of the Isolated Strains for Reducing F. graminearum PH-1 Pathogenicity and DON Production

Potential strains were selected using a dual culture assay on PDA plates. The *F. graminearum* PH-1 spore suspension (10^6^ spores/mL) and the selected bacterial cell suspensions (10 µL of each) were mixed in equal parts (1:1) and cultured on PDA agar plates at 25 °C in the dark for five days. Purification of *F. graminearum* PH-1 from the bacteria was performed, and the purified fungi once again interacted with the bacteria. This process was repeated several times until the isolated bacteria showed significantly reduced virulence of *F. graminearum* PH-1 by measuring colony diameter and color after each interaction step. At least three replicates were considered in each step, and the whole experiment was repeated three times.

The pathogenicity of purified *F. graminearum* PH-1 in wheat coleoptile and flowering wheat heads was determined and conducted as described previously [47]. For the wheat coleoptile infection assay, 2 µL of conidia suspension (10^6^ spore/mL) was inoculated into fresh wheat coleoptile wounds, and disease symptoms were observed six days after incubation. Each treatment contained 36 wheat coleoptiles with 3 replicates. For wheat spikelet infection assay, 10 µL of spore suspensions (10^5^ spore/mL) was inoculated into flowering wheat spikelet, and disease indexes were observed two weeks after incubation in the field. Each treatment contained 21 flowering wheat heads with three replicates. For the DON production assay, the point-of-inoculation wheat spikelet of the wild-type *F. graminearum* PH-1 and purified *F. graminearum* PH-1 were collected, dried, and grounded into fine powders and stored at 4 °C until analysis [48]. Each treatment, as well as the control, had five wheat spikelets with three replicates. 

### 5.4. Biosafety Assessed of Strain GSE25

Hemolytic activity was carried out according to Fu et al. [49]. The LB medium was used to culture the isolated strain GSE25 at 25 °C, and tryptic soy broth medium (TSB) containing 0.5% glucose was used to culture *S. aureus* ATCC 29213 at 37 °C as a positive control. After an overnight culturing period, bacterial cells were harvested, washed twice with PBS buffer, and re-suspended until an OD600 nm = 0.5 (ca. 10^8^ CFU/mL) was reached. Then, 20 µL of cell suspension was spotted and lined onto the sheep blood agar (SBA) plate, and hemolysis zones were measured on the SBA plates after 24 h. Virulence factors such as *estA*, *estB*, *estAB*, and *hla* were investigated. On the other hand, *E. coli* ETEC and *S. aureus* ATCC 29213 [50] served as positive controls. The DNA extraction of strain and PCR analysis of virulence factors were performed according to Lin et al. [19] and Vergara et al. [20].

### 5.5. Plant Pathogenicity Test of Strain GSE25

The cell-free supernatant of the culture medium of GSE25 (CFS) was filtered three times through 0.22 µm filters. After centrifugation, the pallets of GSE25 cells were washed three times with sterile water (BC) and re-suspended at a concentration of OD600 nm = 0.2 (ca. 10^5^ CFU/mL), OD600 nm = 0.3 (ca. 10^6^ CFU/mL), and OD600 nm = 0.5 (ca. 10^8^ CFU/mL), respectively. Fresh wheat coleoptile wounds were inoculated with 2 µL of BC (10^6^ CFU/mL) and CFS separately, and disease symptoms were observed six days after incubation. On the other hand, flowering wheat heads were inculcated with 10 µL of BC (10^6^ CFU/mL) and CFS separately, and the disease index was observed two weeks after incubation in the field. Each treatment contained 36 wheat coleoptiles and 21 flowering wheat heads with at least 3 replicates. At the same time, 1 mL of BC (OD600 nm = 0.2, OD600 nm = 0.3, and OD600 nm = 0.5, respectively) was slowly injected into wild-type *Nicotiana benthamiana* leaves, which were grown in the greenhouse of Northwest A&F University under a 16/8 photoperiod at 25 °C for five weeks. After growing for three days, the degree of damage to leaves (*n* = 5) was observed. To investigate the plant growth-promoting ability of strain GSE25, nine seeds of spring wheat line Fielder were sown in a plastic plot size that contained 200 g of sterile saline soil, and the seeds were covered about 1 cm of soil in a shaded greenhouse at 25 °C during the day and 20 °C at night. Irrigation of the potted seeds was performed every five days for three weeks with 50 mL of BC (10^6^ CFU/mL) and 50 mL CFS. The growth of the plants was observed every week. The experiments were repeated three times, with six replicates per potted plant.

### 5.6. Whole Genome Sequence of Strain GSE25

Genomic DNA was extracted using the TIANamp Bacterial genomic DNA kit (Beijing, China) according to the manufacturer’s protocol. Sequencing libraries were prepared using the NEXTflex rapid DNA-sequencing kit (Bio Scientific Inc., Austin, TX, USA) for paired-end Illumina sequencing (2 × 150 bp) on a NovaSeq 6000 platform (Illumina, San Diego, CA, USA). The library was loaded onto a R9.4 flow cell and sequenced using a PromethION DNA sequencer (ONT, Oxford, UK) for 48 h. The raw Illumina sequencing reads generated from the paired-end library were subjected to quality filtering using fastp v0.23. Nanopore reads were extracted, base called, demultiplexed, and trimmed using ONT Guppy (ONT, Oxford, UK), with a minimum Q score cutoff value of 7.

### 5.7. Phylogenetic Tree Analysis of Strain GSE25

Based on 16 s rRNA gene sequence analysis, whole-genome sequence analysis, and PhyloPhlAn method [51], the evolutionary position of strain GSE25 was identified as belonging to the *Enterobacteriaceae* family. To validate the specificity of this finding, GSE25 genomic reads were assembled onto bacterial genomes of *Enterobacteriaceae* species downloaded from the NCBI microbial genome database. A total of 72 *Enterobacteriaceae* species genome sequences were used to construct a phylogenetic tree (Table S4), which included *Escherichia*, *Klebsiella*, *Enterobacter*, *Kosakonia*, *Cedecea*, *Cronobacter*, *Kosakonia,* and *Pseudescherichia*, of which type strains were used for each genus. According to the methods of Alnajar and Gupta, multiple sequence alignments of the genome were created using Clustal Omega (EMBL-EBI, Cambridgeshire, UK) [23], in which regions in the alignments with gaps present in N50% of genomes were trimmed by Gblocks 0.91 b, and the final trees were visualized using MEGA 6.

### 5.8. DNA-seq and RNA-seq Analyses of Inhibitory Effects of Strain GSE25 on F. graminearum PH-1

High-quality total DNA from the fungal material was extracted using the CTAB method. Total RNA was isolated from hyphae of each sample using the TaKaRa RNAiso Reagent (TaKaRa Biotechnology, Dalian, China), and cDNA libraries were constructed using the NEBNext^®^ UltraTM Directional RNA Library Prep Kit (New England Biolabs, Ipswich, MA, USA) using the manufacturer’s instructions. The DNA and cDNA libraries were sequenced using an Illumina Hiseq Novaseq platform with paired-end reads (150 bp) by Novogene Corporation (Beijing, China) for DNA-seq and RNA-seq analysis, respectively. Reads were mapped to the genome of *F. graminearum* strain PH-1. Salomn and DESeq (version 2) program were used for differential expression transcript analysis by Novogene Corporation (Beijing, China). Transcripts with log2 Fold change >1 or <−1 and *p*-value < 0.05 were corrected using the false discovery rate (FDR). Raw data of the DNA-seq and RNA-seq were deposited in the NCBI under the accession number.

The differential expression transcripts were functionally annotated with GO data using Trinotate 3.2.2 to facilitate BLASTx searches of the transcript coding sequences against the UniProtKB protein database. GO enrichment analysis was carried out with Blast2GO to detect enriched gene ontology terms among up and down-regulated genes, using an FDR < 0.05. To observe the expression of genes related to pathogenicity and DON production, some functionally validated genes (Table S3) and *TRI* gene clusters were screened from differentially expressed genes obtained via RNA-Seq analysis.

### 5.9. In Vitro Antifungal Activity of Strain GSE25 against F. graminearum

The CFS and BC were obtained using the same procedure described above, and BC was re-suspended and diluted to 1 × 10^8^ CFU/mL with 50 mL YEPD medium. A 250 mL culture fluid of GSE25 (CF) was centrifuged at 10,000 rpm for 10 min at 4 °C. Subsequently, BC was re-suspended with 50 mL sterile PBS buffer and ultrasonically broken until the cell lysate (CL) was transparent at 4 °C for 40 min (CL(P)); at the same time, BC was re-suspended using 50 mL YEPD medium and ultrasonically broken was the sample CL(Y). In addition, the CL(P) was treated in two ways: one was heated in a water bath at 60 °C for 15 min (CLH(P)), and the other was treated with 200 µL protease K (100 μg/mL) at 60 °C for 15 min (CLHP(P)). The effect of the different treatment groups on spore germination, germ tube growth, and the mycelia morphology of *F. graminearum* PH-1 was evaluated according to Ibrahim et al. [11]. Briefly, 1 mL spore suspension (10^6^ spores/mL) was added into 50 mL of different treatment fluids, including CF, CFS, BC(Y), BC(P), CL(P), CL(Y), CLH(P), and CLHP(P), with shaking (175 rpm) at 25 °C. At six different action time points (0, 9, 24, 48, 72, and 96 h), 1 mL of liquid cultures of different treatment groups was transferred into a 2 mL sterile Eppendorf tube, centrifuged at 10,000 rpm for 2 min, and observed for spore germination, germ tubes growth, and the mycelial morphology of *F. graminearum* PH-1 using the optical microscope. The experiment was repeated six times, with three replicates for each different treatment. For pathogenicity and DON production assay, 2.5 µL of re-suspended liquid cultures of each treatment were inoculated into fresh wheat coleoptile wounds, and disease symptoms were observed six days after incubation at 25 °C, and 10 µL were inoculated into flowering wheat heads, and the disease index was observed two weeks after incubation in the field. Each treatment contained 36 wheat coleoptiles and 21 flowering wheat heads with three replications. DON concentrations of each treatment were measured as described above. Each treatment had five flowering wheat heads with three replicates.

### 5.10. Biocontrol Effect of Strain GSE25 on FHB under Field Conditions

The experimental design of the field traits was initiated in Yangling, Shaanxi Province. The average temperature of the growing wheat season in 2022 and 2023 was 11–21 °C (April to May) and 20–30 °C (June to July). The experimental plots (15 m^2^) were randomized block designs with nine wheat heads with five replicates per treatment. The cell concentration of CF was adjusted to 1 × 10^8^ CFU/mL using a sterile LB medium, while BC was adjusted to a concentration of 1 × 10^8^ CFU/mL in distilled water. The concentration of spore suspension of *F. graminearum* PH-1 was counted and adjusted to 10^6^ spores/mL. Every nine wheat heads were tied together in a cluster as a parallel, and a total of five treatments were performed as follow: (i) Un-sprayed with 10 mL water as control group; (ii) Sprayed with 10 mL spore suspension of *F. graminearum* PH-1 as positive control group; (iii) CFS treatment group: sprayed with 10 mL CFS, a co-spraying with 5 mL CFS and 5 mL spore suspension of *F. graminearum* PH-1 (mixed in a 1:1 ratio), sprayed with 5 mL CFS for three days and then sprayed with 5 mL spore suspension of *F. graminearum* PH-1, and sprayed with 5 mL spore suspension of *F. graminearum* PH-1 for three days and then sprayed with 5 mL CFS; (iv) BC treatment group: sprayed with 10 mL BC, a co-spraying with 5 mL BC-W and 5 mL spore suspension of *F. graminearum* PH-1 (mixed in a 1:1 ratio), sprayed with 5 mL BC for three days and then sprayed with 5 mL spore suspension of *F. graminearum* PH-1, and sprayed with 5 mL spore suspension of *F. graminearum* PH-1 for three days and then sprayed with 5 mL BC; (v) CF treatment group: sprayed with 10 mL CF, a co-spraying with 5 mL CF and 5 mL spore suspension of *F. graminearum* PH-1 (mixed in a 1:1 ratio), sprayed with 5 mL CF for three days and then sprayed with 5 mL spore suspension of *F. graminearum* PH-1, and sprayed with 5 mL spore suspension of *F. graminearum* PH-1 for three days and sprayed with 5 mL CF. Each treatment was sprayed using a plastic watering attachment, ensuring the liquid was in a mist. After treatment, wheat heads were moisturized with plastic bags for 48 h, and the incidence of each treatment group was measured two weeks after spraying. The final percentages of visibly infected spikelets were classified according to the equation below. The disease severity score was determined as follows: score 0 = no disease symptom, 1 = very slight (<20%), 2 = slight (21–40%), 3 = moderate (41–60%), 4 = severe (61–80%), and 5 = completely infected spikelet [43].

$$\%\;Incidence=\frac{\Sigma (Infected\;plants\;at\;each\;level\times Relative\;level\;value)}{Total\;plants\times 5}\times 100\%$$

### 5.11. Statistical Analysis

The obtained data was processed and analyzed using GraphPad Prism 8.0 (GraphPad Software, San Diego, CA, USA). A one-way analysis of variance test was used to determine the sources of detected significance (*p* < 0.05), followed by a Tukey HSD multiple-comparison test as a post hoc analysis. The data is presented as the mean ± standard deviation (SD).

## Figures and Tables

**Table 1:** Identification of bacterial isolates from wheat seeds based on 16S rRNA gene sequence fragments in the National Center for Biotechnology Information (NCBI) GenBank databases.

Strain ID	Closest Relative (Obtained from BLAST Search)	Identity (%)	Matched GenBank No.
GSE01	*Pseudescherichia vulneris* strain NBRC 102420	100	NR114080
GSE06	*Pseudescherichia vulneris* strain ATCC 33821	99	NR041927
GSE07	*Enterobacter cancerogenus* strain LMG 2693	99	NR044977
GSE08	*Enterobacter hormaechei* subsp. strain 10–17	100	NR126208
GSE09	*Pseudescherichia vulneris* strain NBRC 102420	100	NR114080
GSE10	*Pseudescherichia vulneris* strain NBRC 102420	100	NR114080
GSE12	*Pseudescherichia vulneris* strain NBRC 102420	100	NR114080
GSE14	*Enterobacter bugandensis* strain 247BMC	99	NR148649
GSE15	*Klebsiella grimontii* strain SB73	100	NR159317
GSE16	*Enterobacter cancerogenus* strain LMG 2693	99	NR116756
GSE19	*Enterobacter asburiae* strain JCM6051	98	NR024640
GSE25	*Pseudescherichia vulneris* strain NBRC 102420	100	NR114080
GSE26	*Pseudescherichia vulneris* strain NBRC 102420	100	NR114080
GSE28	*Pseudescherichia vulneris* strain ATCC 33821	99	NR041927
GSE32	*Yokenella regensburgei* strain CIP 105435	99	NR104934
GSE37	*Yokenella regensburgei* strain JCM 2403	100	NR112986
GSE38	*Pseudescherichia vulneris* strain NBRC 102420	100	NR114080
GSE43	*Pseudescherichia vulneris* strain NBRC 102420	100	NR114080
GSE44	*Pseudescherichia vulneris* strain ATCC 33821	99	NR041927
GSE47	*Pseudescherichia vulneris* strain NBRC 102420	100	NR114080

**Table 2:** Quality assessment for strain GSE25 assembly.

Statistics	Number
Clean reads	287,299
Average read length (bp)	12,475
N50 length (bp)	18,768
Genome length (bp)	4,761,311
Coding gene	4486
Total length (bp)	4,208,967
GC (%)	55.6
rRNA	22
tRNA	85

## Data Availability

The data presented in this study are available in this article.

## Supplementary Materials

The following supporting information can be downloaded at: https://www.mdpi.com/article/10.3390/toxins15120702/s1. Figure S1: Colony morphology of *Pseudescherichia* sp. GSE25 on PDA agar; Figure S2: Plant growth-promoting effect of *Pseudescherichia* sp. GSE25. A: Leaf length; B: Root length. Spring wheat line Fielder was used as the wheat cultivar. Significance was determined through ANOVA (ns: no significance; **** *p* < 0.0001); Figure S3: Functional prediction of *F. graminearum* PH-1 interaction with GSE25 based on Nr, eggNOG, GO, and KEGG annotation. A: The species distribution of sequences assembled in the Nr database. B: EggNOG functional classification. C: GO functional classification. D: KEGG metabolic pathway classification; Table S1: General database annotations; Table S2: Annotation of variation gene loci in *Fusarium graminearum* PH-1; Table S3: Some previously reported functionally validated genes in *Fusarium graminearum* PH-1; Table S4: A total of 72 *Enterobacteriaceae* species genome sequences used to construct a phylogenetic tree.
