# Supplementary material for: Novel Endophytic Pseudescherichia sp. GSE25 Strain Significantly Controls Fusarium graminearum and Reduces Deoxynivalenol in Wheat
Source: Toxins (Basel). 2023 Dec 15;15(12):702. doi: 10.3390/toxins15120702 (PMC10747052; doi:10.3390/toxins15120702)
Supplement: Supplementary file 1 [file toxins-15-00702-s001.zip › Figure S3.pdf]

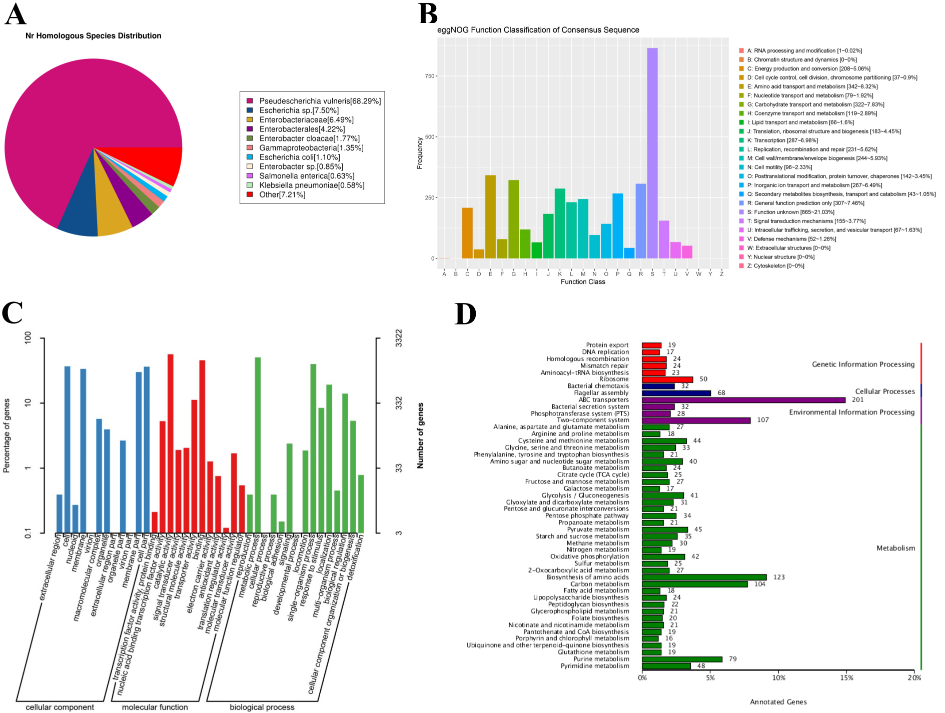
A
Nr Homologous Species Distribution
Pseudescherichia vulneris[68.29%]
Escherichia sp.[7.50%]
Enterobacteriaceae[6.49%]
Enterobacterales[4.22%]
Enterobacter cloacae[1.77%]
Gammaproteobacteria[1.35%]
Escherichia coli[1.10%]
Enterobacter sp.[0.85%]
Salmonella enterica[0.63%]
Klebsiella pneumoniae[0.58%]
Other[7.21%]
B
eggNOG Function Classification of Consensus Sequence
Frequency
Function Class
A: RNA processing and modification [1~0.02%]
B: Chromatin structure and dynamics [0~0%]
C: Energy production and conversion [208~5.06%]
D: Cell cycle control, cell division, chromosome partitioning [37~0.9%]
E: Amino acid transport and metabolism [342~8.32%]
F: Nucleotide transport and metabolism [79~1.92%]
G: Carbohydrate transport and metabolism [322~7.83%]
H: Coenzyme transport and metabolism [119~2.89%]
I: Lipid transport and metabolism [66~1.6%]
J: Translation, ribosomal structure and biogenesis [183~4.45%]
K: Transcription [287~6.98%]
L: Replication, recombination and repair [231~5.62%]
M: Cell wall/membrane/envelope biogenesis [244~5.93%]
N: Cell motility [96~2.33%]
O: Posttranslational modification, protein turnover, chaperones [142~3.45%]
P: Inorganic ion transport and metabolism [267~6.49%]
Q: Secondary metabolites biosynthesis, transport and catabolism [43~1.05%]
R: General function prediction only [307~7.46%]
S: Function unknown [865~21.03%]
T: Signal transduction mechanisms [155~3.77%]
U: Intracellular trafficking, secretion, and vesicular transport [67~1.63%]
V: Defense mechanisms [52~1.26%]
W: Extracellular structures [0~0%]
Y: Nuclear structure [0~0%]
Z: Cytoskeleton [0~0%]
C
Percentage of genes
Number of genes
cellular component
molecular function
biological process
extracellular region, cell, nucleoid, membrane, virion, macromolecular complex, organelle, extracellular region part, organelle part, virion part, membrane part, cell part
protein binding transcription factor activity, nucleic acid binding transcription factor activity, catalytic activity, signal transducer activity, structural molecule activity, transporter activity, binding, electron carrier activity, antioxidant activity, translation regulator activity, molecular transducer activity, molecular function regulator
reproduction, metabolic process, cellular process, reproductive process, biological adhesion, signaling, developmental process, locomotion, single-organism process, response to stimulus, localization, multi-organism process, biological regulation, cellular component organization or biogenesis, detoxification
D
Protein export 19
DNA replication 17
Homologous recombination 24
Mismatch repair 24
Aminoacyl-tRNA biosynthesis 23
Ribosome 50
Bacterial chemotaxis 32
Flagellar assembly 68
ABC transporters 201
Bacterial secretion system 32
Phosphotransferase system (PTS) 28
Two-component system 107
Alanine, aspartate and glutamate metabolism 27
Arginine and proline metabolism 18
Cysteine and methionine metabolism 44
Glycine, serine and threonine metabolism 33
Phenylalanine, tyrosine and tryptophan biosynthesis 21
Amino sugar and nucleotide sugar metabolism 40
Butanoate metabolism 24
Citrate cycle (TCA cycle) 25
Fructose and mannose metabolism 27
Galactose metabolism 17
Glycolysis / Gluconeogenesis 41
Glyoxylate and dicarboxylate metabolism 31
Pentose and glucuronate interconversions 21
Pentose phosphate pathway 34
Propanoate metabolism 21
Pyruvate metabolism 45
Starch and sucrose metabolism 35
Methane metabolism 30
Nitrogen metabolism 19
Oxidative phosphorylation 42
Sulfur metabolism 25
2-Oxocarboxylic acid metabolism 27
Biosynthesis of amino acids 123
Carbon metabolism 104
Fatty acid metabolism 18
Lipopolysaccharide biosynthesis 24
Peptidoglycan biosynthesis 22
Glycerophospholipid metabolism 21
Folate biosynthesis 20
Nicotinate and nicotinamide metabolism 21
Pantothenate and CoA biosynthesis 19
Porphyrin and chlorophyll metabolism 16
Ubiquinone and other terpenoid-quinone biosynthesis 19
Glutathione metabolism 19
Purine metabolism 79
Pyrimidine metabolism 48
Genetic Information Processing
Cellular Processes
Environmental Information Processing
Metabolism
Annotated Genes
