# Supplementary material for: Novel Endophytic Pseudescherichia sp. GSE25 Strain Significantly Controls Fusarium graminearum and Reduces Deoxynivalenol in Wheat
Source: Toxins (Basel). 2023 Dec 15;15(12):702. doi: 10.3390/toxins15120702 (PMC10747052; doi:10.3390/toxins15120702)
Supplement: Supplementary file 1 [file toxins-15-00702-s001.zip › Table S1.pdf]

Table S1. General Database Annotations.

Database	Number	100<=length<300	length>=300
eggNOG_Annotation	4,028	1,851	1,919
GO_Annotation	3,322	1,440	1,697
kegg_Annotation	2,784	1,181	1,475
nr_Annotation	4,454	2,530	1,977
Pfam_Annotation	3,943	1,782	1,898
Swissprot_Annotation	3,312	1,425	1,685
TrEMBL_Annotation	4,448	2,053	1,977
All_Annotated	4,459	2,055	1,977
