# Supplementary figures and images for: Novel Endophytic Pseudescherichia sp. GSE25 Strain Significantly Controls Fusarium graminearum and Reduces Deoxynivalenol in Wheat
Source: Toxins (Basel). 2023 Dec 15;15(12):702. doi: 10.3390/toxins15120702 (PMC10747052; doi:10.3390/toxins15120702)

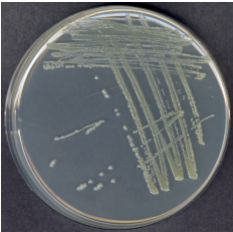

Supplement: Supplementary file 1 [file toxins-15-00702-s001.zip › Figure S1.pdf]

**A**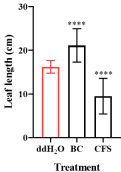**B**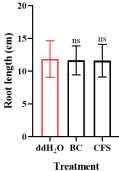

Supplement: Supplementary file 1 [file toxins-15-00702-s001.zip › Figure S2.pdf]
